# Suppressor of fused associates with dissemination patterns in patients with glioma

**DOI:** 10.3389/fonc.2022.923681

**Published:** 2022-08-24

**Authors:** María Peris-Celda, Josefa Carrión-Navarro, Irina Palacín-Aliana, Pilar Sánchez-Gómez, Ricardo Prat Acín, Noemi Garcia-Romero, Angel Ayuso-Sacido

**Affiliations:** ^1^ Department of Neurosurgery, Mayo Clinic, Rochester, NY, United States; ^2^ Faculty of Experimental Sciences, Universidad Francisco de Vitoria, Madrid, Spain; ^3^ Atrys Health, Barcelona, Spain; ^4^ Fundación de Investigación HM-Hospitales, Madrid, Spain; ^5^ Faculty of Science, Universidad de Alcalá, Madrid, Spain; ^6^ Neurooncology Unit, Instituto de Salud Carlos III-Unidad Funcional de Investigación de Enfermedades crónicas (UFIEC), Madrid, Spain; ^7^ Departamento de Neurocirugía, Hospital Universitario La Fe, Valencia, Spain; ^8^ Brain Tumor Laboratory, Fundación Vithas, Grupo Hospitales Vithas, Madrid, Spain; ^9^ Faculty of Medicine, Universidad Francisco de Vitoria, Madrid, Spain

**Keywords:** glioma, brain tumors, SuFu = suppressor of fused, migration, hedgehog glioma-associated oncogene 1 (GLI1), glioblastoma

## Abstract

Gliomas are the most common brain tumors, which present poor prognosis, due, in part, to tumor cell migration and infiltration into distant brain areas. However, the underlying mechanisms causing such effects are unknown. Hedgehog (HH)–Gli axis is one of the signaling pathways involved, with a high number of molecular mediators. In this study, we investigated the association between HH-Gli intermediates and clinical parameters. We found that high levels of SuFu are associated with high dissemination patterns in patients with glioma. Therefore, we analyzed SuFu expression data in three glioma cohorts of surgical samples (N =1,759) and modified its expression in Glioblastoma Cancer Stem Cells (GB CSC) *in vitro* models. Our data reveal that SuFu overexpression increases cancer stemness properties together with a migratory phenotype. This work identifies SuFu as a new molecular player in glioma cell migration and a promising target to develop blocking agents to decrease GB dissemination.

## Introduction

Gliomas are the most common tumors of the central nervous system (CNS) with an incidence of 6.6 per 100,000 habitants, with a higher incidence in men (1). These heterogeneous tumors are divided into three groups by their glial cell composition: astrocytomas, oligodendrogliomas, and ependymomas (2). Tumor grade (I-IV) depends on tumor malignancy and is defined and established by the World Health Organization (WHO) (3). Brain Magnetic Resonance Image (MRI) and histological evaluation are the gold standard techniques for glioma diagnostic (4); nonetheless, molecular analysis has been included in the last 5 years (3). Current glioma medical treatment is based on surgery, radiotherapy, and chemotherapy with the alkylating cytostatic agent temozolomide or with nitrosureas, depending on the tumor grade. The prognosis of patients with glioma is poor and the median overall survival of the most aggressive kind, glioblastoma (GB) (classified as grade IV), is less than 21 months (5). Despite advances in genomics, transcriptomics, and epigenetic characterization (6), few increases in survival rates have been observed in the last decades, highlighting the lack of knowledge about glioma tumor biology.

Tumor initiation, progression, relapse, and, in some cases, even therapy escape mechanisms are driven, in part, by the presence of a small subpopulation of cancer stem cells (CSCs) in the brain (7). CSC invasion capacity of the surrounding brain determines disease progression; however, the effect of this migration pattern in patients’ prognosis is still unclear (8). One of the signaling pathways that has been suggested to be involved in gliomagenesis is the Hedgehog (HH)–Gli axis (9). This signaling cascade is active during the embryogenic development and normally is repressed in adult life, except in some pathologies like cancer, where it can be reactivated (10). Briefly, in humans, the activation of this pathway occurs when sonic HH binds to the transmembrane protein Patched 1 (Ptch1) and liberates Smoothened (Smo), which activates Gli transcription factors (11). The Gli complex is composed of three Gli proteins, normally suppressed by the negative regulator Suppressor of fused (SuFu) (12). Mutations on this gene are associated with some CNS tumors such as medulloblastoma or meningioma (13), and its loss appears to increase tumorigenesis (14). In an attempt to investigate HH-Gli pathway in gliomas, some assays have been developed (15, 16). However, the role of SuFu and its mechanism of action have not yet been elucidated. For this reason, and to investigate SuFu function in different grade glioma tumors, we analyzed its expression in three cohorts (N = 1,759) and established the correlation with migration and stemness patterns. Our data reveals that increased levels of SuFu correlate with high dissemination patterns in patients with glioma and in GB CSCs *in vitro* models.

## Materials and methods

### Human glioma and control samples

A total of 79 surgical samples were obtained from patients diagnosed with glioma. The brain tumors were classified by histology (astrocytoma, N = 10; oligodendrolgioma, N = 6; oligoastrocytoma, N = 1; and GB, N = 62) based on WHO criteria. Surgical procedures and MRI were conducted at Hospital La Fe (Valencia, Spain). All patients gave their informed patient consent in accordance with the medical and science ethics review board.

### Cancer stem cell cultures

GB18 and GB27 CSCs were processed within 12 h of extraction, following the previously described protocol (17). Medium was replaced every 3 days. Sphere-like clusters were passaged before becoming necrotic by both enzymatic and mechanical disaggregation with polished Pasteur pipettes.

### Vector construction

To induce the overexpression of SuFu in the CSCs, SuFu cDNA was PCR-amplified and cloned into the vector pWPI, which contains the GFP sequence following the internal ribosome entry site (**Supplementary Figure 1A**). Lentiviruses were generated by cotransfecting the backbone carrying SuFu along with pCMV AR8.2 and pMD2 VsVg into 293T cells. Transfection was performed using the CalPhos Kit (Calbiochem).

To induce the downregulation of SuFu, an entry vector for the expression of short hairpin RNA (shRNA) was designed with the BLOCK-iT™ U6 Entry Vector Kit (Invitrogen) and was then transfected into 293FT cells using the BLOCK-iT™ Lentiviral RNA Expression System (Invitrogen). The vector containing the shRNA was cloned into the pLenti6/BLOCK-iT™ -DEST vector that contains Blasticidin resistance marker for the selection of infected cells (**Supplementary Figure 1B**). The corresponding empty vector was used as an internal control (shRC-).

Viral particles from both lentiviral systems were collected after 72 h. The viral particles were then purified by ultra-centrifugation at 26,000 rpm for 90 min at 4°C and resuspended in media. These viruses were used to infect CSCs with the use of Lipofectamine™ 2000 (Invitrogen).

### Development of infected CSCs

After infection with the upregulating lentiviral vector, CSCs were sorted by green fluorescent protein (GFP) expression using a MoFlo High Speed Cell Sorter (Beckman-Coulter). One cell per well was plated by Fluorescent Activated Cell Sorter (FACS)-automatic cell deposition in 96-well plates and allowed to expand clonally with the media being replaced every other day. The clone with the highest GFP expression, as confirmed by immunocytochemistry (**Supplementary Figure 2**), was used for all experiments. The level of GFP correlated with the level of transgene expression. However, with each successive passage, diminishing levels of either GFP or transgene were observed. To minimize variations in the transgene expression in subsequent passages, all experiments were carried out within the first 10 passages after clonal selection. As for the cells infected with the downregulating vector, blasticidin (4 µg/ml) was used to select the infected cells. The antibiotic was kept in the media for at least 10 days, and a booster dose was given every couple of passages.

### RNA extraction and cDNA synthesis

RNA extraction from tissues and CSCs was performed with TriReagent (Sigma) following the manufacturer’s recommendations. RNA concentration was measured with a NanoDrop 2000 Spectrophotometer (Thermo Fisher Scientific). RNA was reverse-transcribed with the High-Capacity cDNA Reverse Transcription Kit (Applied Biosystems).

### qRT-PCR

Resulting cDNA was diluted and analyzed by quantitative real-time PCR (qRT-PCR) using the Light Cycler 1.5 (Roche) sequence detection system with the SYBR Premix Ex Taq (Takara). Primers were designed using the Primer 3 software. Conditions were as follows: one cycle at 95°C for 10 min, followed by 45 cycles of 10 s at 95°C, 10 s at the primer hybridization temperature and 10 s at 72°C. 2^−△△Ct^ method was adopted to analyze the qRT-PCR results. Three housekeeping genes (Glyceraldehyde-3-phosphat dehydrogenase (GAPDH), β2-microglobuline, and β-actin) were used to normalize the data. All data are expressed as increased/decreased folds (relative units). Two replicates were set for each sample, and each reaction was repeated to ensure reproducibility. The information related to all the primers used can be found in **Supplementary Table 1**.

### Cell-surface adhesion assay

Adhesion assays were carried out using different types of surfaces for optimum cell growth. Cell suspensions (2 × 10^4^ cells per well) were seeded onto different extracellular matrix (ECM) components such as Matrigel™ (BD Biosciences), Laminin (Sigma-Aldrich), Fibronectin (Sigma-Aldrich), Gelatin (Sigma-Aldrich), synthetic Poly-D-Lysine (BD Biosciences), and Poly-L-Ornithine (Sigma-Aldrich)–coated surfaces.

Furthermore, polystyrene culture plates (BD Biosciences) were included as controls. Cells were cultured for 48 h, and then, images were taken with an Axiovert 40 CFL Carl Zeiss, microscope using a 20× objective.

### Immunocytochemistry

All immunocytochemistry was performed on CSCs cultured in chamber slides and fixed with 4% paraformaldehyde (PFA) for 15 min at RT. Blocking was then performed with PBS with 2% BSA and 0.2% Triton X-100 for 1 h at RT. Primary antibodies mouse monoclonal anti-vinculin (1:300, Sigma-Aldrich), Alexa Fluor 647- Phalloidin (1:40, Invitrogen) and rabbit anti-SuFu (1:250, Abcam) were incubated overnight and 1 h at RT respectively. Secondary antibodies Alexa 488 anti-mouse (1:500 Invitrogen) and Alexa 488 anti- rabbit (1:500, Molecular Probe) were incubated for 1 h and nuclei were stained with DAPI (1:5000 Sigma-Aldrich). Slides were mounted with fluorescence mounting medium (Fluoromount™ Aqueous Mounting Medium, Sigma-Aldrich).

Fluorescence was observed under a Leica TCS SP5 - Inverted Confocal microscope, equipped with four laser lines to detect immunohistochemical signals. Each confocal micrograph consisted of 1024 (X) × 1024 (Y) x 16 (Z) pixels and all parameters were kept constant. Regions of Interest (ROIs) were defined, and quantification analysis was obtained with Leica Application Suite Advanced Fluorescence (LAS AF) software. The experiment was performed twice, with three images of the different type of cells taken in each event. Negative controls with the secondary antibodies were carried out in all cases.

### Directional changes

5,000 CSCs per well were seeded on a Matrigel-coated 96-well plate. Changes in direction were tracked using an HCS IN Cell Analyzer 1000 (Cytiva, UK).

### Spheres formation

After tumor sphere dissociation, CSCs were plated in 96-well plate at a density of 100 cells per well. One week later, spheres were counted and photographed. Sphere measures were obtained using ImageJ.

### Cell viability assay

For cell viability assay, 3000 cells per well were plated in triplicates for each group (GB18wt, GB18SuFu and GB18sh5) on 96-well plates. Cell proliferation reagent MTS (CellTiter 96 Aqueous One Solution Reagent (Promega)) was added into wells and incubated for 3h at 37°C. Then, absorbance was measured at 490 nm and 630 nm to subtract background and non-specific absorbance in a plate reader (Varioskan Flash Multimode Reader, ThermoScientific). These experiments were performed four times and by triplicate for each time. Cell viability was expressed as percentage of viable cells.

### Migration assays

To evaluate cell migration, a 24-well plate with 8-μm pore size polycarbonate membrane inserts was used. Cells were resuspended in 100 μl serum-free DMEM medium and seeded in the upper chamber, and 500 μl of media with growth factors was added to the lower chamber. After 3 h, migrated cells were fixed with 4% PFA and stained with 0.1% crystal violet. All experiments were performed in triplicate.

### Analysis of SuFu expression in publicly available glioma datasets

GlioVis data portal (http://gliovis.bioinfo.cnio.es) for visualization and analysis of brain tumor expression datasets was used to analyze gene expression data (18). Processed transcriptomic data from publicly available data cohorts [The Cancer Genome Atlas (TCGA) (N = 667) and Chinese Glioma Genome Atlas (CGGA) (N = 1013)] were used.

### Statistical analysis

Quantitative variables were tested for normality using the Saphiro test. Under normally distributed conditions, mean differences were inferred using the two tailed t-test. The chi-squared test and Fisher’s exact test were used to compare independent variables.

Statistical tests were performed using SPSS software version 20. The clinical variables studied were revised on three separate occasions for each patient and included an independent external review to minimize the error possibility.

Differences between groups were considered statistically significant when p < 0.05.

## Results

### High level of SuFu is associated with a high dissemination pattern in patients with glioma

With the aim of obtaining an overview of the HH-Gli pathway in glioma, we examined the association between pre-operative MRI clinical parameters and HH-Gli intermediates expression in tumoral tissue. Among the comparison test statistics, we found that the most significant corresponds to higher SuFu levels associated with increased dissemination at the time of diagnosis (p < 0.01) (**Table 1**).

**Table 1:** Comparison of clinical parameters among themselves and its association with HH-Gli intermediates expression.

Clinical Parameters			
Comparison	Significance	Interpretation	
Tumor size–distance to ventricle (< or > 5 mm)	0.007	Higher tumor size was related with less distance to the ventricle	
Mortality at 6 months after resection	0.009	More mortality →̲lower resection	
Dissemination through white matter tracts-Resection	0.007	More dissemination →̲lower resection	
Mortality at 6 months–distance to ventricle	0.04	More mortality →̲less distance	
Mortality at 6 months–dissemination through white matter tracts	0.039	More mortality →̲high dissemination	
**HH-Gli intermediates expression and clinical parameters**			
**Comparison**	**Significance**	**Interpretation**	
Smo–tumor size	0.034	Higher Smo levels are associated to higher tumor size	
SuFu–dissemination at diagnostic time	0.01	Higher SuFu overexpression is associated to increased dissemination	
Glioma grade–Gli1 expression	0.016	Higher Gli1 levels are found in LGG^1^	
Glioma grade–Gli1/SuFu	0.041	Higher Gli1/SuFu ratio in LGG	
Glioma grade–Smo/Ptch1	0.038	Higher Smo/Ptch1 ratio in LGG	

^1^LGG, low-grade glioma. Significant data (p < 0.05) are shown.

**Figure 1:**
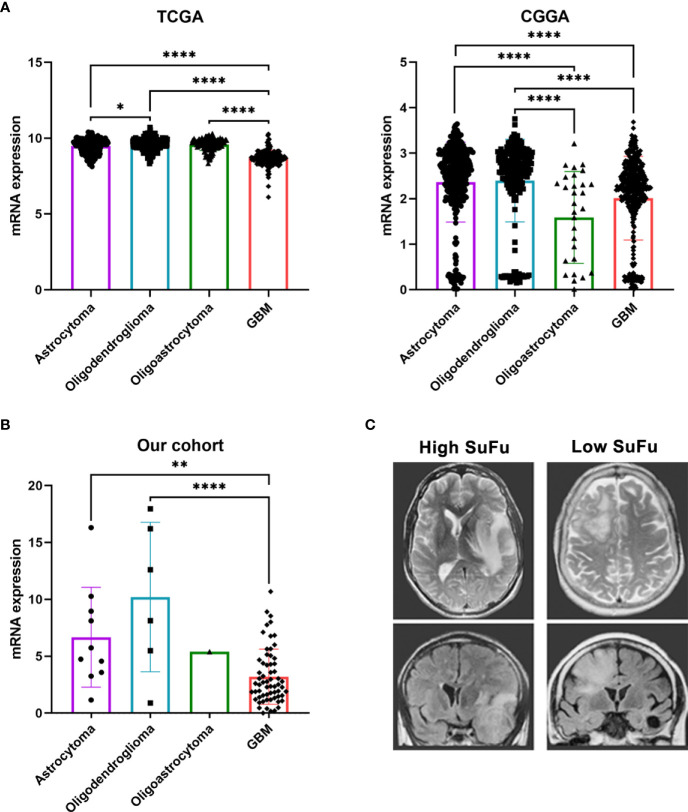
**(A)** SuFu mRNA expression analysis in TCGA (N = 667) and CGGA (N = 1,013) gliomas cohort, grouped according to histological type. **(B)** Quantitative RT-PCR analysis of SuFu in our cohort (N = 79). **(C)** Illustrative example of MRI, T2 axial (upper), and coronal T2 Flair Sequence right (bottom). *P < 0.05, **P < 0.01 and ****P < 0.0001.

For this reason, we decided to focus our study on elucidating SuFu’s role in glioma tumors. An *in silico* study, as shown in **Figure 1A**, of the SuFu transcriptomic data obtained from TCGA (N = 667) and CGGA (N = 1,013) cohorts revealed that GB have lower SuFu expression levels than LGG. Then, we sought to examine SuFu expression in an external cohort. As presented in **Figure 1B**, we observed two defined subpopulations divided in high and low expression levels of SuFu. In addition, we visualized matched MRI images from the two most extreme datapoints, the two patients with the highest and the lowest SuFu. **Figure 1C** illustrates the differences between high (left) and low (right) grade of white matter infiltration, corresponding to SuFu gene expression.

### SuFu upregulates stemness without an effect in CSCs growth/proliferation

**Figure 2:**
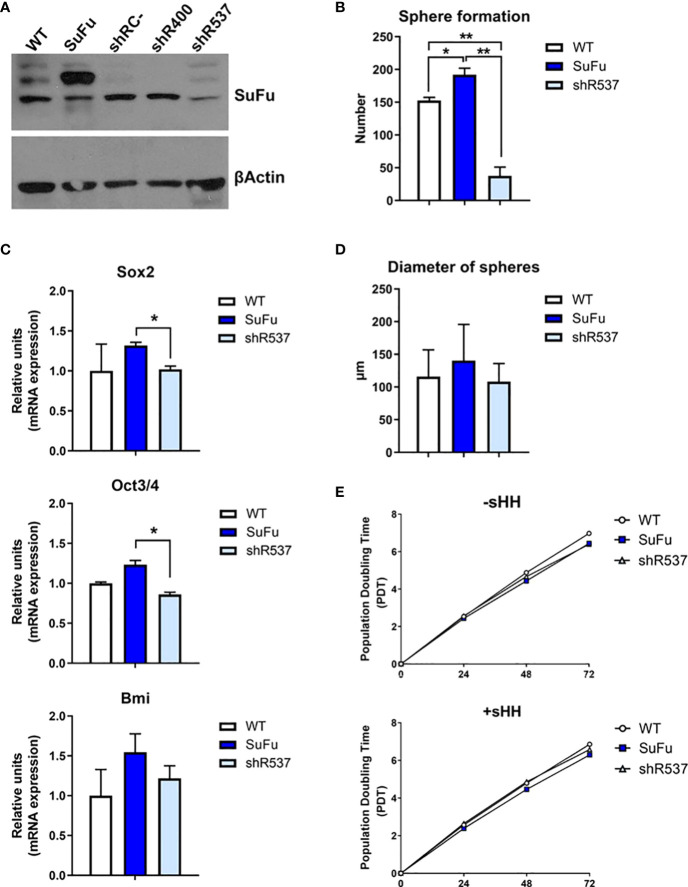
**(A)** Western blot analysis for SuFu using GB18 CSCs samples transfected with overexpression and downregulation vector. CSCs transfected with empty expression vector (shRC-) was used as control. **(B)** Number of spheres formed by GB18 CSCs **(C)** *Sox2*, *Oct3/4*, and *BMI* mRNA levels in GB18 CSCs by qRT-PCR. **(D)** Diameter of spheres of GB 18CSCs. **(E)** Duplication time in basal conditions (−sHH) and in the presence of ligand (+sHH). WT, SuFu, and shR537 CSCs were analyzed. Data are shown as mean ± S.D. and are representative of two independent experiments. P-values were calculated based on the two-tailed two-sample t test. *P < 0.05 and **P < 0.01.

To examine the role of SuFu in gliomas, we induced its upregulation and downregulation in GB18 and GB27 CSCs. In an attempt to increase efficacy of downregulation, we tested two vectors (shR400 and shR537) and used the shR537 for the next assays due to its higher potency (**Figure 2A** and **Supplementary Figure 3A**). Then, we compared stemness and proliferation properties *in vitro*. SuFu overexpression resulted in increased spheroid formation (**Figure 2B** and **Supplementary Figure 3**) with a higher expression of pluripotency transcriptional factors, including *SOX2*, *OCT3/4*, and *BMI-1* (**Figure 2C**). These results led us to explore the effect of SuFu in GB CSCs proliferation. We first monitored spheres growth by measuring their diameter after a week in culture and no differences were noticed among groups, as shown in **Figure 2D** and **Supplementary Figure 3**.

Second, we added Sonic-Hedgehog (Shh) recombinant protein to the cell culture media, as it is one of the ligands that activate the Hedgehog signaling pathway. The three CSCs showed similar population doubling time (PDT) values (**Figure 2E**). Altogether, by the increased expression of pluripotency markers and sphere formation counts, these results suggest that SuFu is involved in GB CSC stemness potential.

### SuFu expression influences cell–cell and cell–matrix adhesion protein profile

**Figure 3:**
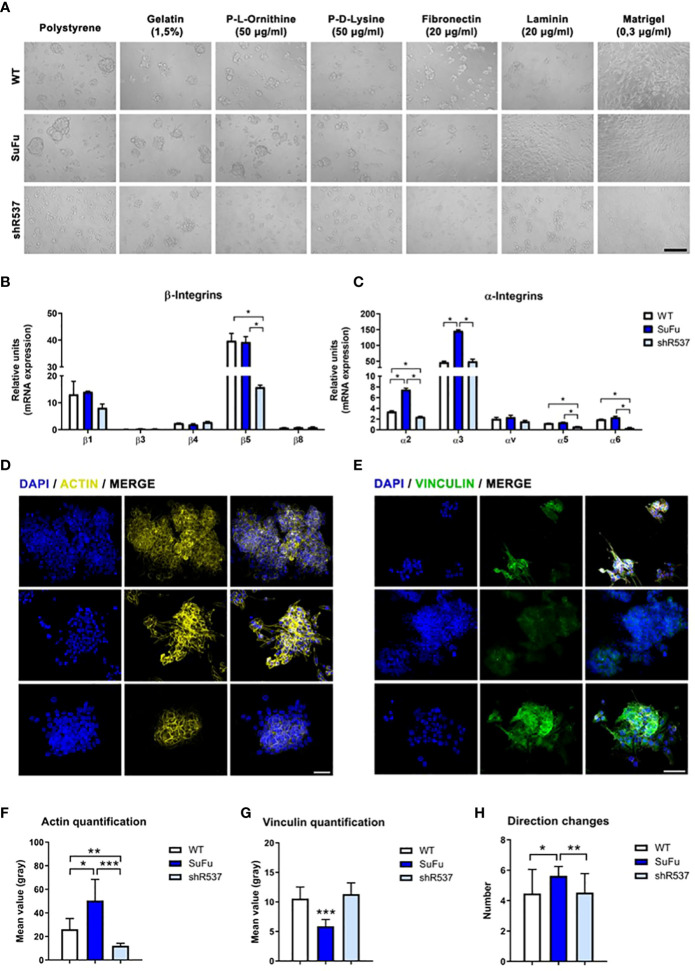
**(A)** Cell adhesion assay *in vitro*. **(B)** β-integrin subunits expression in GB18 CSCs. **(C)** α-integrin subunits expression in GB18 CSCs. **(D)** Representative image of actin immunofluorescence staining. **(E)** Immunofluorescence staining of vinculin. Scale bar represents 50 µm. **(F)** Immunofluorescence quantification of actin protein. **(G)** Immunofluorescence quantification of vinculin protein. **(H)** Movement of the cells measured by number of directional changes. *P < 0.05, **P < 0.01 and ***P < 0.001.

To interrogate about the involvement of SuFu mRNA levels in cell–cell and cell–ECM interactions, we performed an adhesion assay using different ECM compounds. As observed in **Figure 3A**, we noticed higher adhesion in the SuFu overexpressed cell line in all materials analyzed. As these adhesive interactions are mostly mediated by the associated α and β transmembrane subunits of the integrins, we evaluated their expression. All β integrins expression levels were similar for each cell line except for the β5 subunit which was downregulated in the shRNA cell line (**Figure 3B**). On the contrary, all the α subunits were highly upregulated in the SuFu cell line (**Figure 3C**). These observations correlate with the adhesion patterns observed in the previous assays, because α3 and α5 bind fibronectin and α6 engages laminin which, together with collagen IV, are the main compounds of the Matrigel (19, 20).

As transmembrane integrins play a key role between cell and ECM interaction, we then investigated actin and vinculin cytoskeleton protein expression.

The quantification of fluorescence intensity showed a positive correlation between SuFu expression and actin levels, whereas SuFu upregulation decreases vinculin expression in GB CSCs (**Figure 3D**–**G**). These changes seem to also affect the number of directional changes performed by CSCs in culture (**Figure 3H**).

### SuFu overexpression increases migratory phenotype in GB CSCs

**Figure 4:**
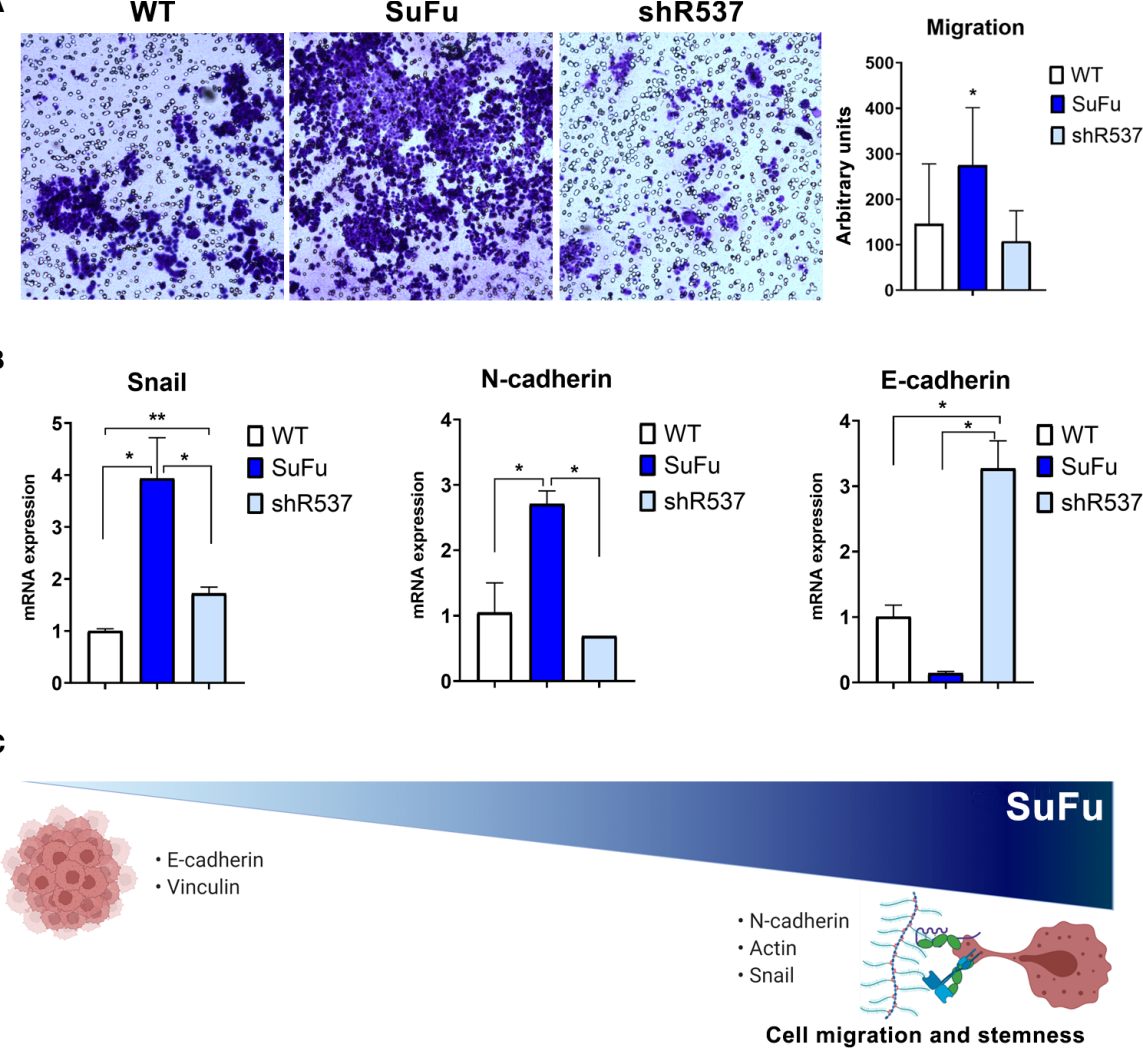
SuFu increases migration and mesenchymal phenotype. **(A)** Transwell migration assay. Relative migration of CSCs quantified with ImageJ. **(B)** Epithelial-to-mesenchymal transition expression genes. Data are shown as mean ± S.D. and are representative of two independent experiments. **(C)** Representative image of SuFu involvement in EMT. P-values were calculated based on the two-tailed two-sample t test. *P < 0.05 and **P < 0.01.

The migration pattern was further confirmed by an *in vitro* transwell assay (**Figure 4A**). The number of migrating CSCs increased when SuFu was overexpressed in comparison with the other CSCs lines (p < 0.05) (**Figure 4B**). As it has been proved that the epithelial–mesenchymal transition (EMT) process contributes to the migration of GB tumors, we decided to evaluate several EMT-related markers (21). Consistent with previous observations, SuFu overexpression induces the transcription factor *Snail*, upregulates the expression of the mesenchymal marker *N-cadherin*, and downregulates *E-cadherin* levels (**Figure 4C**). All these changes seem to be induced by SuFu.

## Discussion

The most prominent hallmarks of GB are its enhanced cell migration and invasiveness. Despite extensive research, the CSC ability to disseminate along the brain parenchyma presents an impediment for maximal surgical resection and radiotherapy (22). Unfortunately, the underlying mechanisms behind these processes still present a challenge for therapy effectiveness.

Cancer cell migration depends on an interplay between several factors and ECM compounds, and normally, correlates with the malignancy grade (23). We and others have demonstrated the association between high dissemination pattern, lower resection, and, consequently, a detriment in the overall postoperative survival (24–26). Elucidating the mechanisms through which glioma cells spread from the original site would lead to novel blocking compounds that could improve clinical outcomes (27).

In our cohort, SuFu levels appear to influence dissemination status at diagnostic time, which has been proposed as a factor related with stemness in GB and other tumors (28). In fact, our results indicate that the overexpression of SuFu upregulates GB stem cell and self-renewal properties without affecting proliferation rates. Although the dichotomy between migration and proliferation has generated intense controversy in glioma field (29), here, we found that both processes present mutually exclusive behaviors when we modulate SuFu expression. The “go or grow” phenomenon is observed in our CSCs with a tendency to the invasive phenotype.

Specifically, some canonical stem pathways such as Hedgehog, Wnt, and Notch play a key role in stemness regulation (30). In accordance with our attachment assay results, this stem phenotype has been previously accepted as a factor required for cell–cell and cell–ECM adhesion (31). The regulation of these interactions is mediated by the αβ heterodimeric transmembrane receptors called integrins (32). Although the interplay between SuFu and those molecules is still unknown, our experiments suggest that SuFu overexpression results in an incremental cell-mediated binding.

Several studies described that traction forces and focal adhesion generated by cells determine cell migration through the ECM (33, 34). In this sense, vinculin, in which its main function apart from focal adhesion creation is actin cytoskeleton regulation (35), is one of the most relevant molecules involved. In other tumors, such as breast, colorectal, and rhabdomyosarcomas cancer, it has been reported that loss of vinculin increases cell migration and correlates with poor prognosis (36–38). The dissemination pattern observed in our SuFu overexpressed population appears to be related to a loss in vinculin and indirect upregulation of actin. The indicated effect has been observed in lung cancer, suggesting that it can directly enhance EMT (39), a fact that could be also influenced by β integrin activation, reducing E-cadherin and upregulating N-cadherin (40).

Although gliomas are not considered tumors with an epithelial origin, it has been widely accepted that EMT has an essential role in their development and spread (41). Our results indicate that SuFu is a key inductor of EMT in GB CSCs. Accordingly, it has been shown that EMT starts when cells lose their adhesions and then promote the tumor dissemination (42). The data presented here showcases that SuFu overexpression is linked to the ability of CSCs to undergo EMT and stemness. However, discordant results have been observed in the relation between EMT and stemness in other tumors, such as pancreas and breast cancer (43, 44). As, sometimes, mRNA abundance may not have a linear relationship with the translated protein expression level, further proteomic experiments are needed to corroborate these findings.

To the best of our knowledge, there is only one published study in which they attempted to elucidate the role of SuFu in gliomas. In addition, although they observed that SuFu ectopic expression restrains cell proliferation and invasion (15), they performed their experiments in established glioma cell lines with compromised stem properties, whereas our assays present a more realistic 3D model (45).

On the basis of our findings, we propose that SuFu could be used as an invasiveness prognosis biomarker, and it could be a promising target to develop blocking agents to decrease GB aggressiveness and dissemination. Indirectly, these treatments would in fact improve surgical resection efficacy by limiting dissemination through parenchyma. Even if these results are promising, in the future, we would need larger cohorts and further investigation to classify SuFu as a tumor dissemination molecular mediator.

## Data availability statement

The raw data supporting the conclusions of this article will be made available by the authors, without undue reservation.

## Ethics statement

The studies involving human participants were reviewed and approved by Hospital Universitario la Fe. The patients/participants provided their written informed consent to participate in this study.

## Author contributions

Conceptualization: MP-C and AA-S. Methodology: NG-R and JC-N. Investigation: NG-R, JC-N, and IP-A. Writing—original draft preparation: NR. Writing—review and editing: NG-R, JC-N, and RPA. Supervision: AA-S. Funding acquisition: AA-S. All authors have read and agreed to the published version of the manuscript. All authors contributed to the article and approved the submitted version.

## Funding

This research was funded by grants from the “Proyectos de Investigación en Salud” (PI21/01353) and La Universidad Francisco de Vitoria-Banco Santander (UFV2021-23).

## Acknowledgments

We are especially grateful to Dr. Francisco Montes (Professor of Statistics and Operative Research at the University of Valencia) for his independent external review of the statistical analysis.

## Conflict of interest

The authors declare that the research was conducted in the absence of any commercial or financial relationships that could be considered as a potential conflict of interest.

## Publisher’s note

All claims expressed in this article are solely those of the authors and do not necessarily represent those of their affiliated organizations, or those of the publisher, the editors and the reviewers. Any product that may be evaluated in this article, or claim that may be made by its manufacturer, is not guaranteed or endorsed by the publisher.

## Supplementary material

Supplementary Figure 1 Vector construction to induce SuFu overexpression **(A)** and downregulation **(B)**.

Supplementary Figure 2 SuFu immunofluorescence was achieved in GB 18.

Supplementary Figure 3 A. Western blot for SuFu using GB27 CSCs samples transfected with shRNA. Non-transfected cells were used as control. B. Number and diameter of spheres formed by GB27 CSCs.

Supplementary Table 1 Primer forward and reverse sequences.
