# Supplementary material for: Suppressor of fused associates with dissemination patterns in patients with glioma
Source: Front Oncol. 2022 Aug 24;12:923681. doi: 10.3389/fonc.2022.923681 (PMC9450955; doi:10.3389/fonc.2022.923681)
Supplement: Supplementary Figure 1 — Vector construction to induce SuFu overexpression (A) and downregulation (B). [file DataSheet_1.docx]

Supplementary Material

# Supplementary Figure 1

**
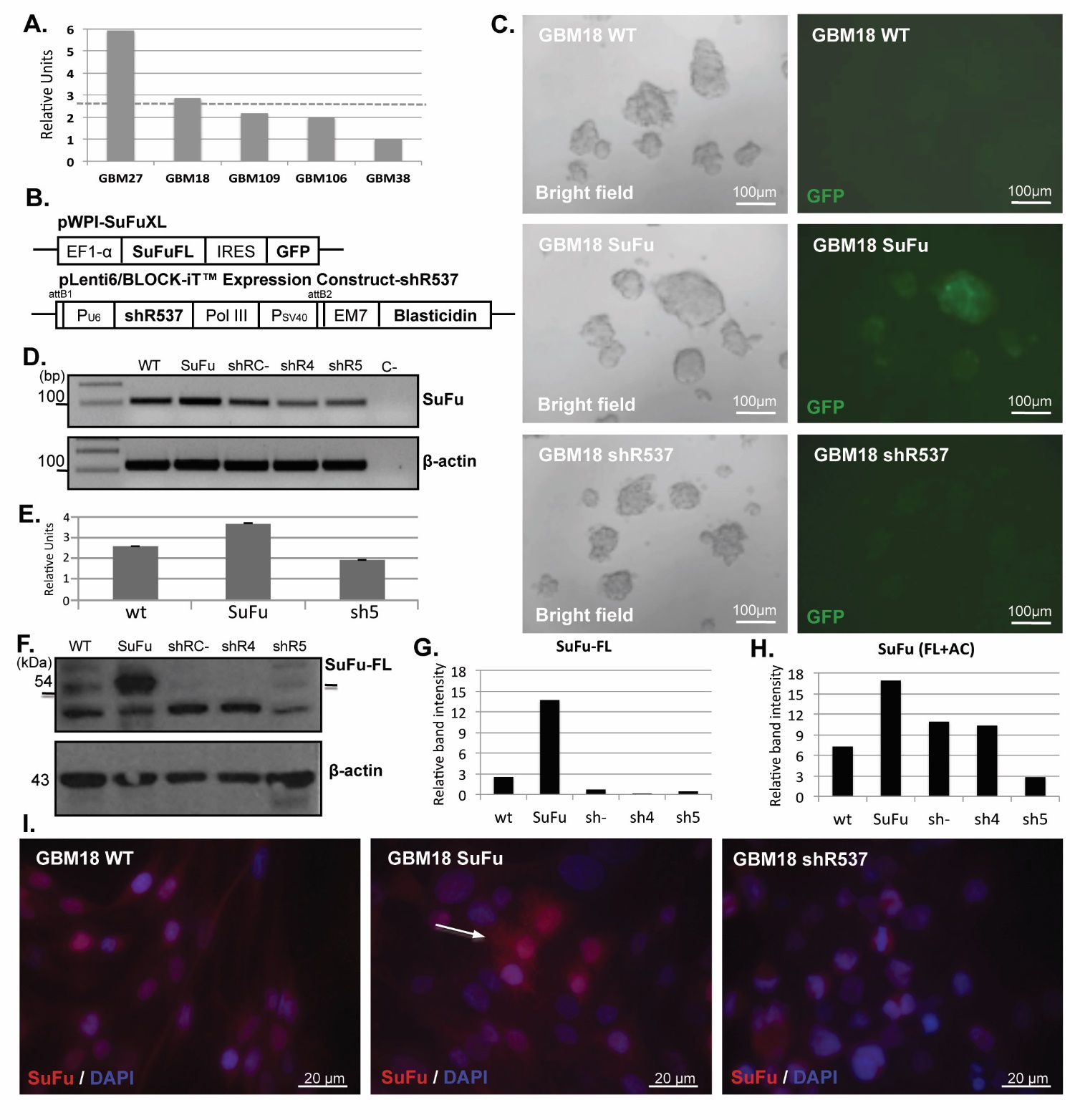
A**


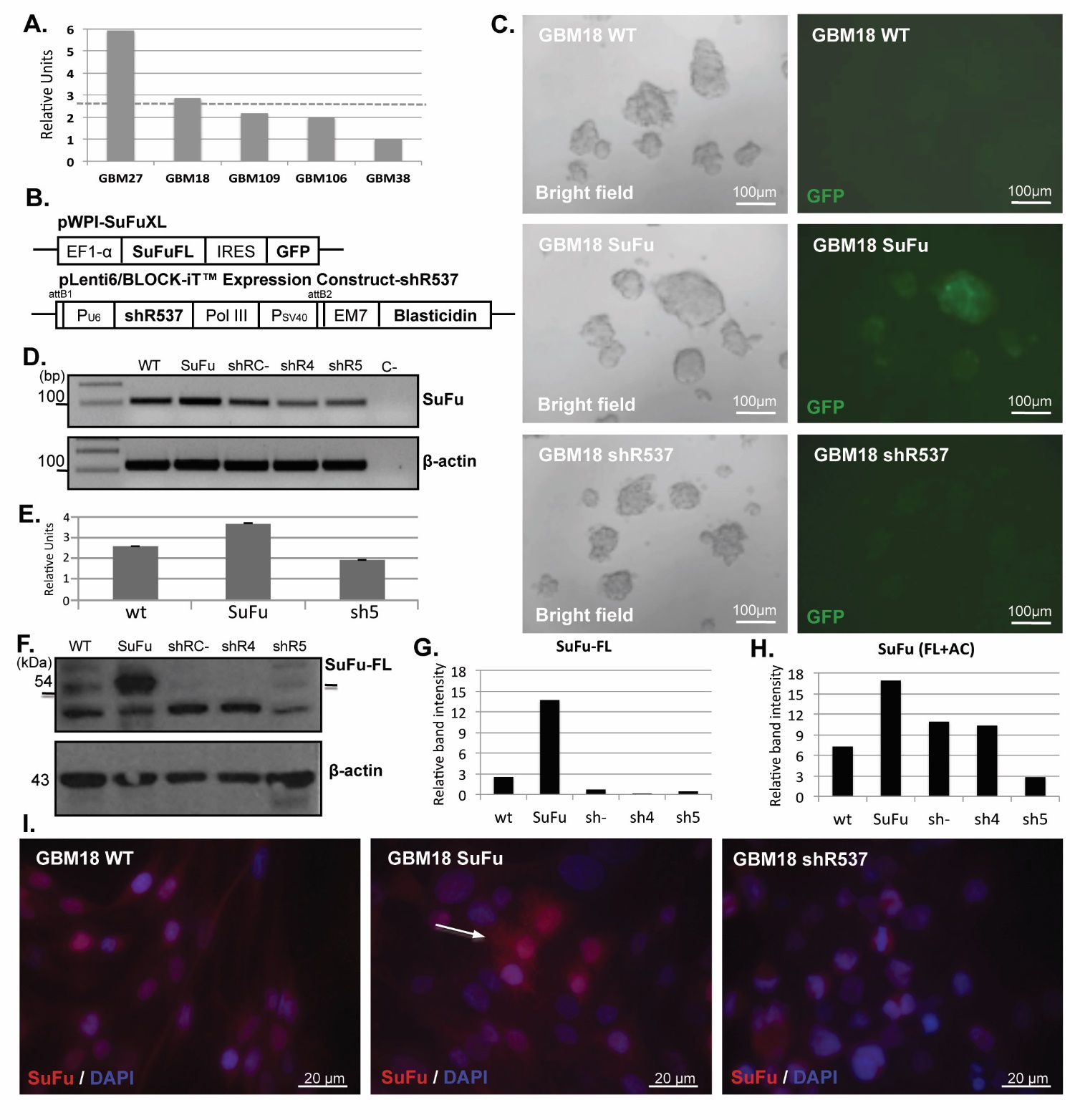
**B**

**Supp. Fig 1.** Vector construction to induce SuFu overexpression (A) and downregulation (B).

# Supplementary Figure 2


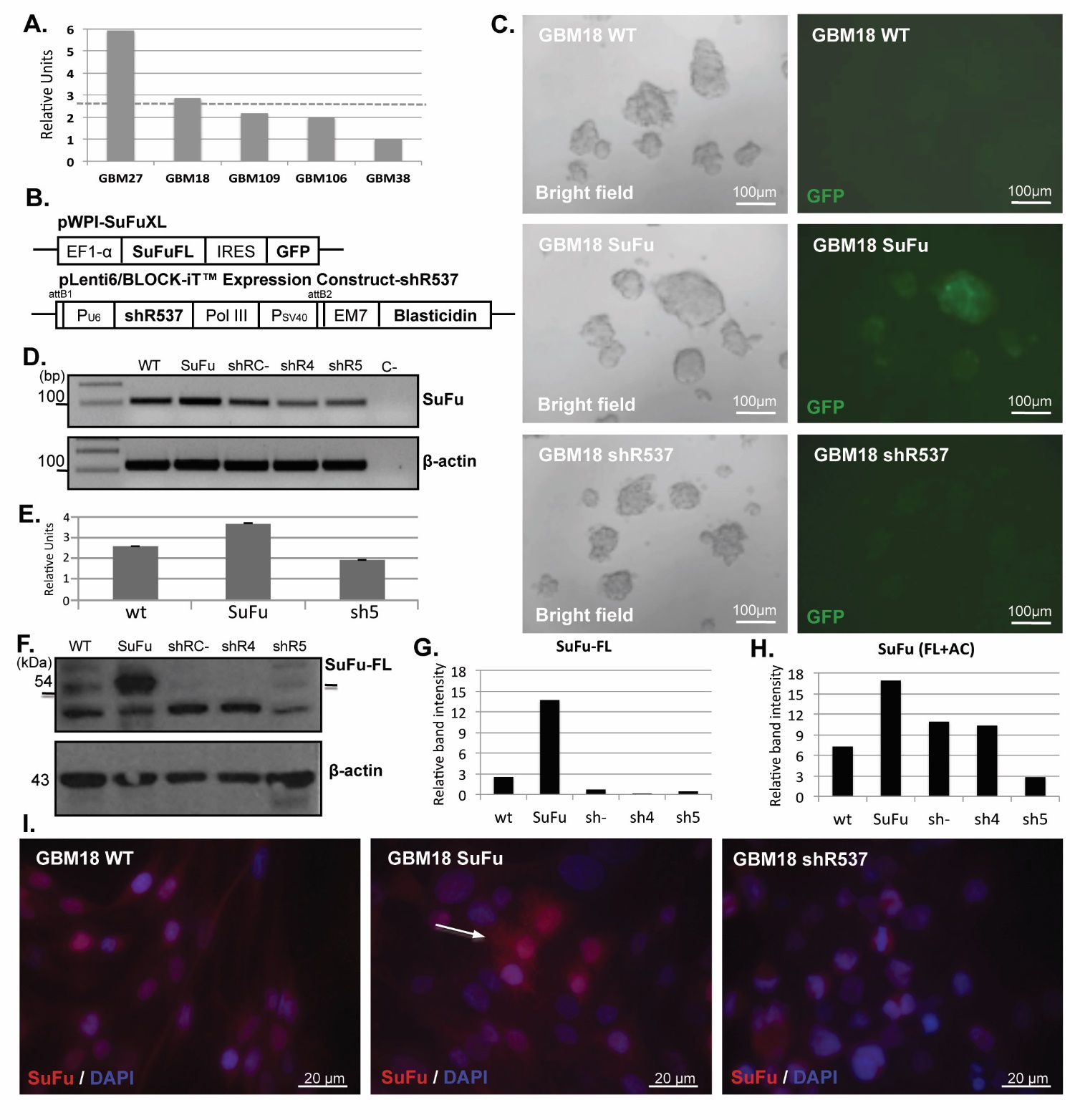

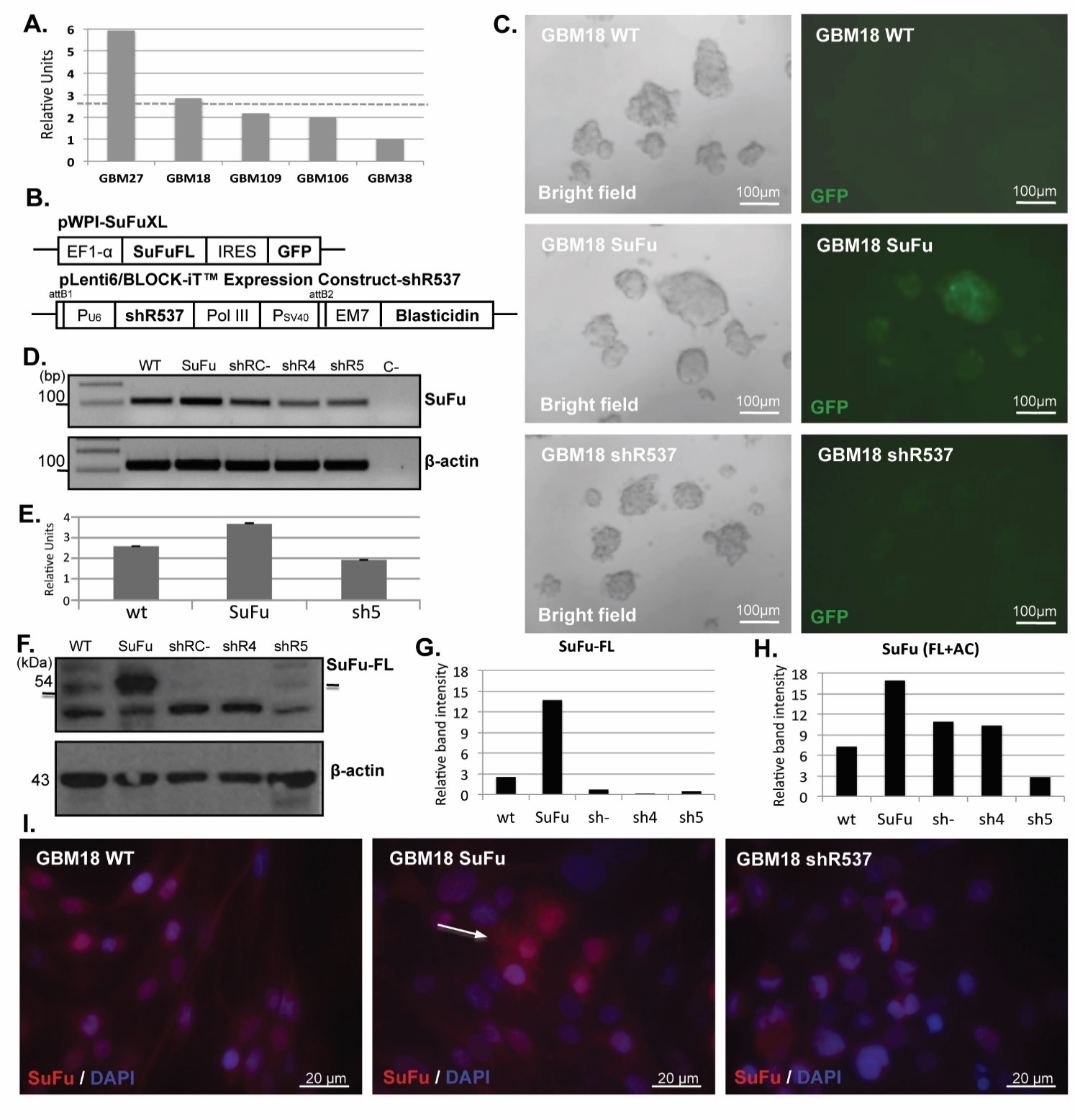

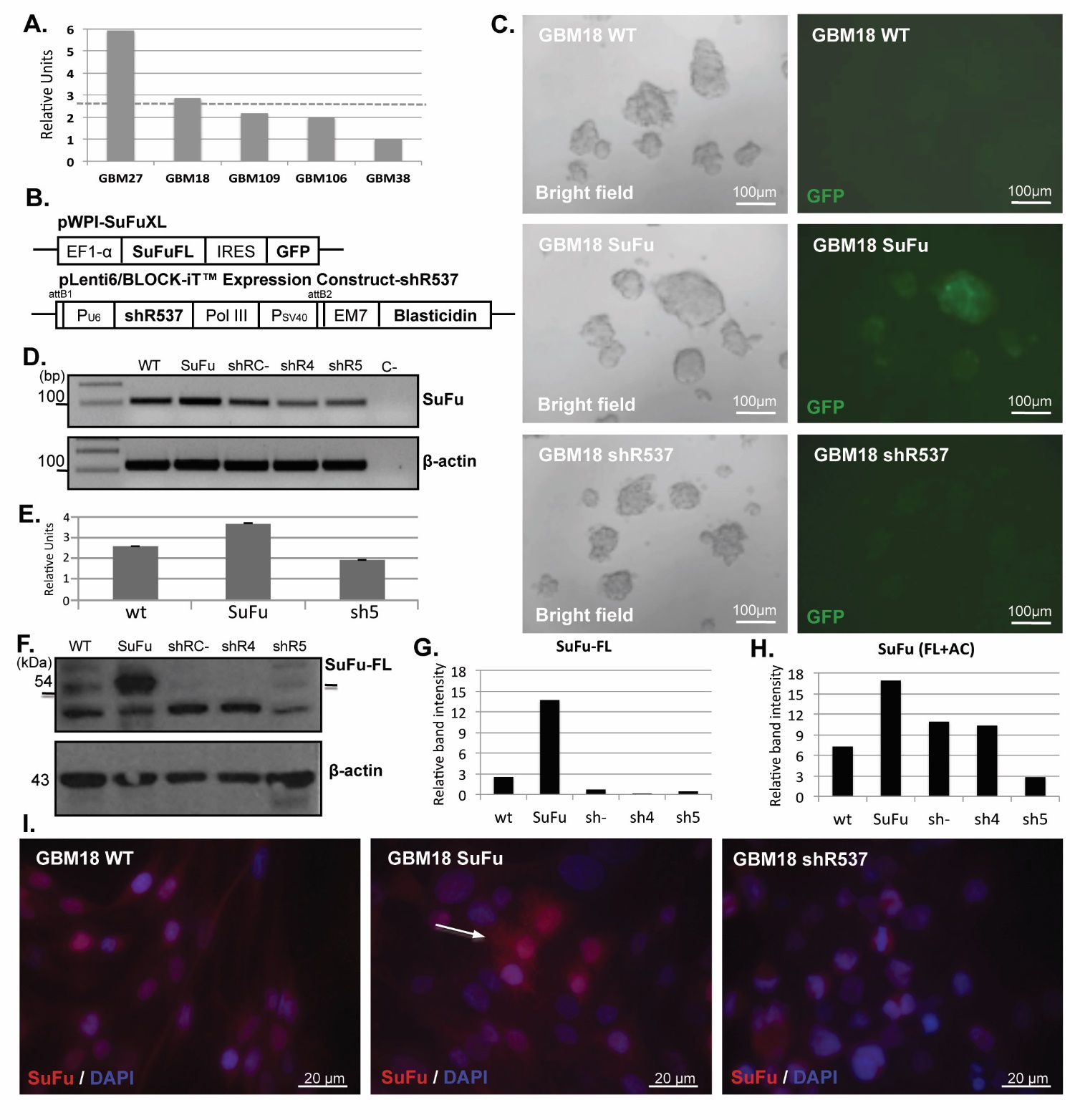


**Supp. Fig 2.** SuFu immunofluorescence was achieved in GB 18. Cells were observed with a Leica DMI4000 B inverted microscope, and pictures were taken with a Leica DFC340FX camera.

# Supplementary Figure 3









**Supp. Fig 3.** A. Western blot for SuFu using GB27 CSCs samples transfected with shRNA. Non-transfected cells were used as control. B. Number and diameter of spheres formed by GB27 CSCs**.**

**Supplementary Table 1.** Primers sequence

| **Gene** | **Forward Sequence 5’🡪3’** | **Reverse Sequence 5’🡪3’** |
| --- | --- | --- |
| *SuFu* | CTAGGACATCCATGCCAGGT | CTTGTCGCAGCATTCAGGTA |
| *Smo* | CCTTCCATAGCCTCCAAACA | TGTATTTCTTCTCCCCATAACCTG |
| *Gli1* | CCAGCCAGAGAGACCAACAG | CCCGCTTCTTGGTCAACTT |
| *Ptch1* | CCCCACTGAAAGATGTAGGC | TAACAAGGTGCTGGTGTGGA |
| *SOX2* | GCACATGAACGGCTGGAGCAACG | TGCTGCGAGTAGGACATGCTGTAGG |
| *OCT3/4* | CAAAAACCCTGGCACAAACT | CCTGTCTCCGTCACCACTCT |
| *BMI-1* | GGAGACCAGCAAGTATTGTCCTTTTG | CATTGCTGCTGGGCATCGTAAG |
| *Integrin β1* | GTTACACGGCTGCTGGTGTT | CTACTGCTGACTTAGGGATC |
| *Integrin β3* | GATGCGAAAGCTCACCAGTA | GCAAGCAGGTGGTCTTCATA |
| *Integrin β4* | AACGATGAACGGTGCCACCT | CTCCACGATGTTGGACGAGT |
| *Integrin β5* | AGGATGCACTGCATTTGCTG | TCCACCGTTGTTCCAGGTAT |
| *Integrin β8* | ATGACGGAAACTGTCATCTG | AGCAATGGTGCCTGGCAAGA |
| *Integrin α2* | CACTCGATTTGGTTCAGCAA | GAACCACTTGTCCAAAGGCA |
| *Integrin α3* | GCCAGCATTGGTGACATCAA | GAATAGCCGAAGGTGGCCAA |
| *Integrin αV* | AGATCTGGACCAGGATGGTT | ATCTGTGGCTCCTTTCATTG |
| *Integrin α5* | CCCGAGTACCTGATCAACCT | GCACACCAGCAACAAAGTCT |
| *Integrin α6* | CAAGATGGCTACCCAGATAT | CTGAATCTGAGAGGGAACCA |
| *SNAIL* | CTTCTCACTGCCATGGAATTCCCT | TCCACAGAAATGGCCATGGGAAA |
| *N-Cadherin* | TCCTATGAGTGGAACAGGAACGCT | AATTGGGGTCTGGAGTTTCGCA |
| *E-Cadherin* | GAGGAATCCAAAGCCTCAGGTCAT | TCACCCACCTCTAAGGCCATCTTT |
| *GAPDH* | TCCTCCACCTTTGACGCTG | ACCACCCTGTTGCTGTAGCC |
| *β2-microglobulin* | CTCGCGCTACTCTCTCTTTCTG | GCTTACATGTCTCGATCCCACT |
| *Β-actin* | TCAAGATCATTGCTCCTCCTGAG | TCAAGATCATTGCTCCTCCTGAG |

Supplementary Table 2. Dissemination at diagnosis time. 1=Yes, 2=No.

| **Patient ID** | **SuFu levels** | **Dissemination** |
| --- | --- | --- |
| 1 | 3,03371167 | 2 |
| 2 | 10,2715098 | 1 |
| 3 | 6,83350413 | 2 |
| 4 | 1,46987965 | 2 |
| 5 | 16,3079103 | 1 |
| 6 | 2,29013986 | 2 |
| 7 | 1 | 1 |
| 8 | 4,68010846 | 2 |
| 9 | 3,24596163 | 2 |
| 10 | 0 | 2 |
| 11 | 8,91290103 | 1 |
| 12 | 5,64057229 | 2 |
| 14 | 7,75915034 | 1 |
| 15 | 8,95230143 | 2 |
| 16 | 1,84166925 | 2 |
| 17 | 8,11262371 | 1 |
| 19 | 7,1107805 | 1 |
| 20 | 1,93499862 | 2 |
| 21 | 4,22556126 | 2 |
| 22 | 16,2011467 | 2 |
| 23 | 5,40324321 | 2 |
| 24 | 2,77780966 | 2 |
| 25 | 4,28138124 | 1 |
| 26 | 4,34247186 | 2 |
| 27 | 1,54196261 | 2 |
| 28 | 8,00861113 | 1 |
| 29 | 3,03846484 | 1 |
| 30 | 2,3470582 | 2 |
| 31 | 1,17054904 | 2 |
| 32 | 1,56484275 | 1 |
| 33 | 8,53541814 | 1 |
| 34 | 6,00716272 | 2 |
| 35 | 2,71350261 | 2 |
| 36 | 5,13905771 | 1 |
| 37 | 1,86429819 | 2 |
| 38 | 4,81500693 | 2 |
| 39 | 3,64974644 | 1 |
| 40 | 2,28236042 | 2 |
| 41 | 2,76759253 | 2 |
| 42 | 1,20580013 | 2 |
| 43 | 1,39417165 | 2 |
